# Anoikis resistance regulates immune infiltration and drug sensitivity in clear-cell renal cell carcinoma: insights from multi omics, single cell analysis and *in vitro* experiment

**DOI:** 10.3389/fimmu.2024.1427475

**Published:** 2024-06-17

**Authors:** Xiangyang Wen, Jian Hou, Tiantian Qi, Xiaobao Cheng, Guoqiang Liao, Shaohong Fang, Song Xiao, Longlong Qiu, Wanqing Wei

**Affiliations:** ^1^ The Department of Surgery, Shenzhen Longgang Second People’s Hospital, Shenzhen, China; ^2^ Department of Urology, The University of Hongkong-Shenzhen Hospital, Shenzhen, China; ^3^ Department of Bone & Joint Surgery, Peking University Shenzhen Hospital, Shenzhen, China; ^4^ Department of Urology, Lianshui People’s Hospital of Kangda College Affiliated to Nanjing Medical University, Huaian, China

**Keywords:** renal cell carcinoma, anoikis, immune microenvironment, prognosis, signature

## Abstract

**Background:**

Anoikis is a form of programmed cell death essential for preventing cancer metastasis. In some solid cancer, anoikis resistance can facilitate tumor progression. However, this phenomenon is underexplored in clear-cell renal cell carcinoma (ccRCC).

**Methods:**

Using SVM machine learning, we identified core anoikis-related genes (ARGs) from ccRCC patient transcriptomic data. A LASSO Cox regression model stratified patients into risk groups, informing a prognostic model. GSVA and ssGSEA assessed immune infiltration, and single-cell analysis examined ARG expression across immune cells. Quantitative PCR and immunohistochemistry validated ARG expression differences between immune therapy responders and non-responders in ccRCC.

**Results:**

ARGs such as CCND1, CDKN3, PLK1, and BID were key in predicting ccRCC outcomes, linking higher risk with increased Treg infiltration and reduced M1 macrophage presence, indicating an immunosuppressive environment facilitated by anoikis resistance. Single-cell insights showed ARG enrichment in Tregs and dendritic cells, affecting immune checkpoints. Immunohistochemical analysis reveals that ARGs protein expression is markedly elevated in ccRCC tissues responsive to immunotherapy.

**Conclusion:**

This study establishes a novel anoikis resistance gene signature that predicts survival and immunotherapy response in ccRCC, suggesting that manipulating the immune environment through these ARGs could improve therapeutic strategies and prognostication in ccRCC.

## 1 Introduction

Clear-cell renal cell carcinoma (ccRCC) represents the most prevalent subtype of renal carcinoma, accounting for approximately 75% of all kidney cancer cases (1). Surgical intervention and chemotherapy currently dominate the therapeutic landscape for this malignancy. Despite a relatively high overall survival rate associated with ccRCC, the occurrence of metastasis in advanced stages drastically reduces the five-year survival rate to below 8% (2). Due to the high recurrence rate and poor prognosis of kidney cancer, it is crucial to inhibit the distant metastasis of renal tumor cells. Whereas tumorigenesis and metastasis are closely related to changes in the tumor microenvironment and the migration ability of tumor cells (3).

Anoikis, a programmed cell death, is triggered by the loss of interactions between cells and the extracellular matrix (ECM) (4). In normal cells, these interactions are disrupted by molecules that initiate anoikis on the cell surface and by glycosylated ECM proteins, leading to apoptosis and cell death. The ECM confines tumor cells to a fixed site within the tissue. Tumor cells that acquire migratory capabilities and move to vascular sites develop resistance to anoikis, allowing them to metastasize to distant locations via the bloodstream, thus forming metastatic foci (5–7). Recent studies have uncovered molecular pathways and mechanisms that regulate resistance to anoikis, including cell adhesion molecules, growth factors, and signaling pathways that induce epithelial-to-mesenchymal transition (8). Downstream molecules in these pathways, such as PI3K/AKT (9) and ERK1/2 (10), play significant roles in apoptosis resistance and survival promotion. The latest research indicates that the Hippo pathway and collagen XIII are linked to anoikis resistance in breast cancer (11, 12).

T cells in the body perform surveillance functions, identifying and eliminating abnormal cells, thereby restricting the survival of tumor cells. The role of immune cell infiltration in shaping the tumor microenvironment and influencing tumor progression has been well recognized (13, 14). Numerous studies have highlighted the impact of immune cell apoptosis on the development and progression of various malignancies, including lung, breast, and endometrial cancers. For instance, research by K. Planells et al. suggested that silencing FAIM2 can inhibit the survival and drug resistance by regulating T cells (15). Additionally, the influence of L1CAM on the prognosis of endometrial cancer has been associated with its role in promoting Treg infiltration, thus impairing resistance to apoptosis (16). While existing research has elucidated the link between immune cell apoptosis and the prognosis of various cancers (17, 18), tumor cells can evade immune detection by acquiring resistance to anoikis (19). Although clinical treatments for kidney cancer include radical surgical interventions, chemotherapy and immunotherapy, there is still a lack of recognized and reliable standard predictors for the diagnosis and prognosis of early-stage kidney cancer. The relationship between immune cells and anoikis, as well as the impact of anoikis on the survival of ccRCC patients, has been minimally explored. Exploring the abnormal performance of immune cells and anoikis within renal cancer tissues holds the potential to uncover new molecular biomarkers that could enhance the accuracy of renal cancer diagnosis and prognosis assessment.

In this study, we developed a prognostic model related to anoikis that stratifies ccRCC patients into different risk categories. Through multi-omics and single-cell analyses, we elucidated the relationship between anoikis and immune cell infiltration across various risk groups. To gain insight into the role of anoikis in cancer immunotherapy, we further explored its relevance to the tumor microenvironment. We investigated its relationship with various immune processes and factors, including immune cell infiltration, immunosuppressive factors, and immunostimulatory factors. Moreover, using quantitative real-time PCR (qRT-PCR) and immunohistochemistry (IHC), we validated the expression patterns of four anoikis-related genes in ccRCC patients responding to immunotherapy. This reveals the potential role of anoikis in influencing the efficacy of immunotherapy and provides novel targets for immunotherapeutic strategies.

## 2 Materials and methods

### 2.1 Data collection process

**Figure 1:**
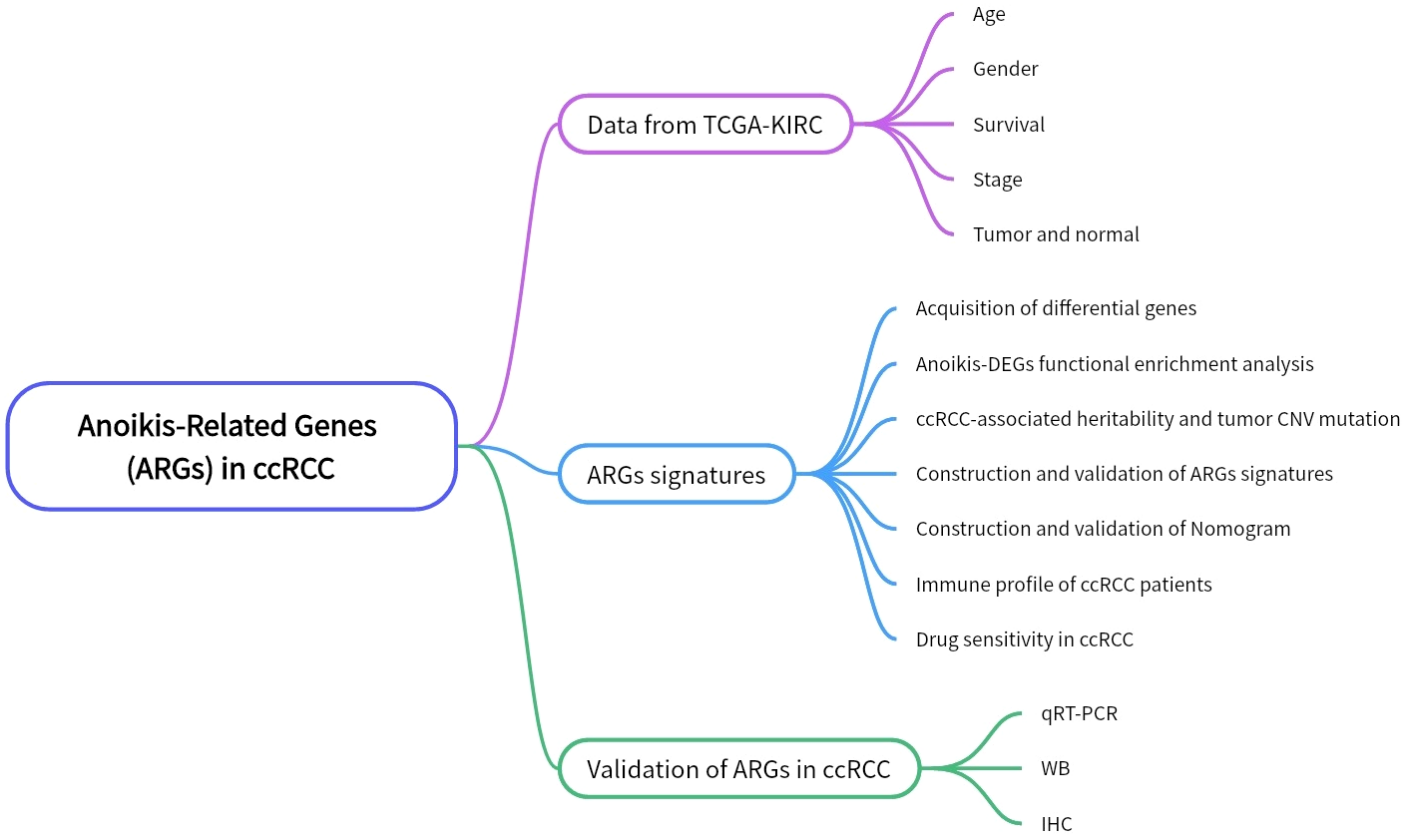
The workflow of this study.

The **Figure 1** shows the flowchart of this study. The “TCGAbiolinks” R package was utilized to retrieve transcriptional data for clear cell renal carcinoma from the TCGA database (TCGA-KIRC; http://cancergenome.nih.gov). We downloaded the data of 542 ccRCC tumor tissues and 72 adjacent non-tumoral tissues. While the chi-square test was applied to compare the clinical characteristics of the two data sets to ensure that random matching did not bias the distribution of clinical characteristics. Anoikis-related genes (ARGs) were sourced from the GeneCards database (https://www.genecards.org/), resulting in the acquisition of 358 ARGs. Single-cell data and a validation cohort for ccRCC transcriptomes were procured from the GEO database.

### 2.2 Differential ARGs identification

We performed differential gene expression analysis on the TCGA-KIRC dataset (including tumor and adjacent non-tumor RNA transcriptomes) using the limma software package to identify differentially expressed genes (DEGs) (P < 0.05) (20). An intersection with the 358 ARGs yielded a subset of differential ARGs for subsequent analyses.

### 2.3 Anoikis functional enrichment

The R packages “GSVA” (21) and “GSEABase” (22) were employed to perform enrichment analysis on the DEGs. The focus of the analysis was to identify the enrichment levels of the anoikis-DEGs on KEGG (Kyoto Encyclopedia of Genes and Genomes) and GO (Gene Ontology) (23). In addition, we have applied Cytoscape software (24) and the STRING database (https://cn.string-db.org/) to explore the protein-protein interactions (25).

### 2.4 Construction of anoikis-related prognostic risk model

First, we selected core prognostic anoikis-related genes (ARGs) using the SVM-RFE algorithm (26). Then, we evaluated the relationship between ARG expression levels and survival in ccRCC patients with univariate Cox regression analysis. Further, we constructed a prognostic model using LASSO Cox regression, utilizing the R packages “survival” and “forestplot”. Subsequently, multivariate Cox proportional hazards regression analysis was conducted to identify critical clinical phenotypes.

The risk score was calculated as the sum of the products of the coefficients and the expression levels of core ARGs (27). We categorized patients into high-risk and low-risk groups based on ARGs-Riskscore median value. In addition, we performed principal component analysis (PCA) mix with Kaplan-Meier analysis to investigate the relationship between the anoikis-based risk scoring and overall survival of ccRCC patients (28). The accuracy of the predictive model was further evaluated using ROC curves. At last, we applied univariate-multivariate Cox regression to validate the predictive power of this risk score model.

### 2.5 Construction and validation of nomogram

Using multivariate Cox and stepwise regression, we incorporated age, TMN staging, and risk scores to construct a prognostic Nomogram to predict overall survival in patients with ccRCC (27, 29). Calibration plots and decision curve analysis (DCA) were constructed to confirm the model’s efficacy and clinical relevance (30). We evaluated the prognostic utility of this model sequentially in the training and test cohorts and in the entire TCGA dataset. This evaluation employed receiver operating characteristic (ROC) curves to assess 1, 3, and 5-year survival predictions (31). Independent prognostic determinants were delineated through sequential univariate and multivariate Cox regression analyses (32), which considered risk scores derived from patient age, gender, and comprehensive TNM staging. The development and assessment of the nomogram were facilitated by the “rms” package in R, ensuring robust discrimination and calibration capabilities within the training dataset.

### 2.6 Immune profile of ccRCC patients based on anoikis resistance

On the basis of the expression of the anoikis-related genes (ARGs), we divided ccRCC patients into two groups: high-risk and low-risk. Then we used the ssGSEA approach to profile the cellular composition of the tumor microenvironment (TME) in the two risk groups (33), while matrix scores and immune scores were assessed to identify differences between these categories. Spearman’s analysis was employed to correlate immune cell characteristics with risk scores. And we examined the immune profiles of all patients through various computational techniques (including cibsort, timer, abs, quantitative, XCELL, and EPIC) (34, 35). Finally, we employed the ssGSEA methodology to assess the immune landscape and scrutinized checkpoint molecules to highlight differences between two groups.

### 2.7 Single-cell analysis of ccRCC based on ARGs

We processed single-cell RNA sequence data using the protocol of the “Seurat” software package (version 4.0.5), while gene expression levels were normalized using the LogNormalize method. Subsequent analysis involved clustering cells and applying t-distributed Stochastic Neighbor Embedding (t-SNE) to identify cellular subpopulations. Our next studies focused on the expression of four anoikis-related genes (ARGs) in ccRCC immune cell subpopulations, while we used a “CellChat” to study intercellular communication between macrophages and dendritic cells.

### 2.8 Clinical ccRCC samples collection

Tissue specimens from ccRCC patients who underwent immunotherapy were acquired from the Department of Urology at the University of Hong Kong Shenzhen Hospital during the period from January 2022 to January 2024. Identifiable details concerning the origins of these tissues were excluded, and the Ethics Committee of the hospital granted approval for this research.

### 2.9 cDNA production and PCR analysis

All cells were acquired from Procell Life Science & Technology Co., Ltd., and maintained in Dulbecco’s Modified Eagle Medium (DMEM) supplemented with 10% fetal bovine serum (FBS) and 1% Penicillin-Streptomycin. The culture conditions were set at 37°C with 5% CO2 (36, 37). Cells at a density of 5×10^5 were plated in six-well plates and incubated for 48 hours. Subsequently, cellular lysis was performed using TRIzol (Invitrogen). RNA was then extracted using a Total RNA Kit. A spectrophotometer was employed to assess the concentration and purity of the RNA. Following this, cDNA synthesis was conducted using an mRNA Reverse Transcription Kit (Roche). Finally, the quantification of target gene expression was achieved by employing a SYBR Green RNA Kit as per the manufacturer’s instructions (38).

### 2.10 Immunohistochemistry

Immunohistochemistry (IHC) was used to validate differential expression levels of anoikis-related genes (ARGs) in ccRCC clinical samples. First, the ccRCC tissue sections were deparaffinized with xylene and washed stepwise with ethanol to rehydrate them. These sections were then treated with 3% hydrogen peroxide for 15 minutes to inhibit endogenous peroxidase activity, followed by antigen retrieval using 1 mM EDTA. Subsequently, the sections underwent overnight incubation at 4°C with antibodies against the model genes, diluted at a ratio of 1:200 (SANTA CRUZ). Following this, we applied PolyHRP Anti-Mouse/Rabbit IgG Detection System (Solarbio, China) and visualized the proteins using diaminobenzidine. Hematoxylin was used for counterstaining before the sections were dehydrated. The prepared slides were examined under a Zeiss microscope. For quantitative analysis, the staining intensity was measured and analyzed using ImageJ and GraphPad Prism version 7 software. Statistical significance was established at P-value < 0.05.

### 2.11 Statistical analysis

Statistical analyses in this study got executed utilizing the R software (release 4.0.2, https://www.r-project.org). Student’s t-test was applied to calculate the DEGs. Comparisons of overall survival (OS) was performed using Kaplan–Meier analysis coupled with log-rank testing. P < 0.05 was established for statistical significance.

## 3 Results

### 3.1 Molecular insights into ARGs regulation and prognostic significance in ccRCC

**Figure 2:**
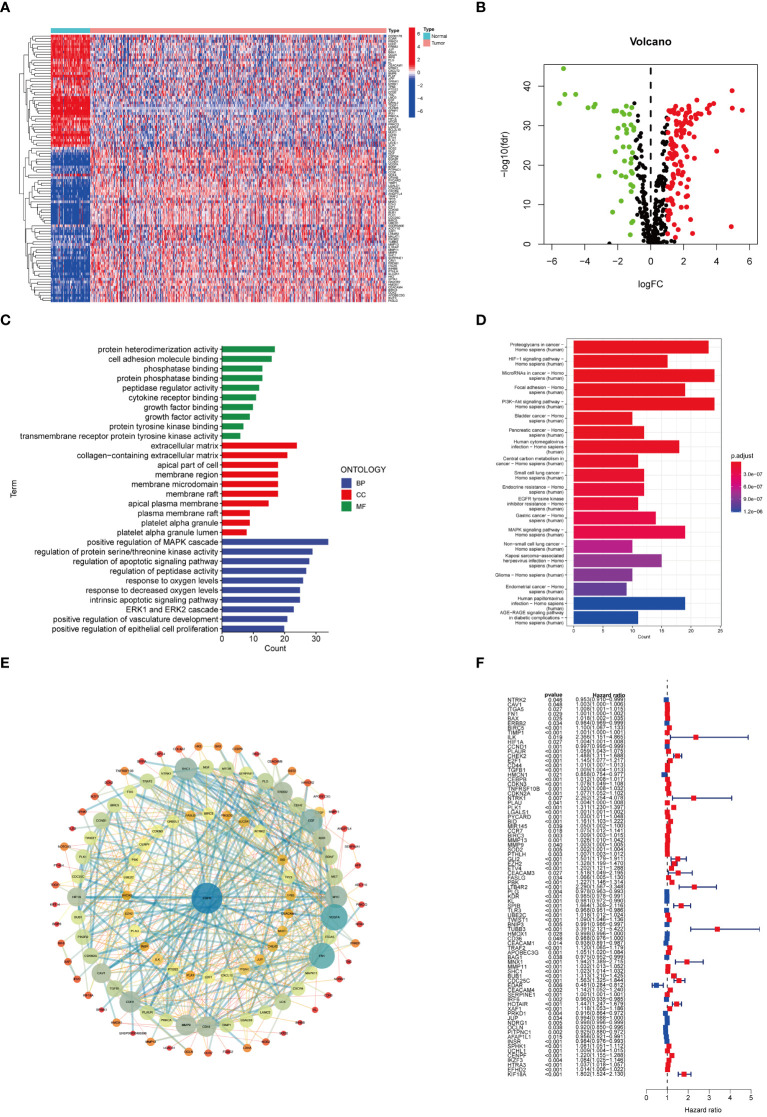
ARGs expression patterns in ccRCC. **(A)** Heat map of ARGs expression. **(B)** Volcano plot of ARGs differential genes. **(C)** Enrichment analysis. **(D)** KEGG analysis based on ARGs. **(E)** DEGs of ARGs. **(F)** Univariate Cox analysis on ARGs.

The workflow of this study is depicted in **Figure 1**, with a comprehensive methodology described in the Methods section. Initially, transcriptome data and clinical information of 161 ARGs were extracted from the TCGA-KIRC cohort. Analysis revealed that 118 ARGs were upregulated, whereas 43 genes showed downregulation. Expression patterns of these differentially expressed genes are visualized in heatmaps and volcano plots (**Figures 2A, B**). Functional enrichment analysis indicated that the majority of ARGs are involved in processes such as the extracellular matrix, positive regulation of the MAPK cascade, regulation of the apoptotic signaling pathway, and the ERK1 and ERK2 cascade (**Figure 2C**), aligning with current insights into the mechanisms of anoikis resistance. Additionally, the HIF-1 signaling pathway and the PI3K-Akt signaling pathway were implicated in this context (**Figure 2D**). A protein interaction network further identified EGFR as a key upstream signaling molecule (**Figure 2E**). Finally, significant correlations between 81 ARGs and the prognosis of ccRCC were identified, with 23 ARGs serving as potential biomarkers for favorable prognosis (**Figure 2F**).

### 3.2 Anoikis genetic epigenetics and prognostic biomarkers in ccRCC

**Figure 3:**
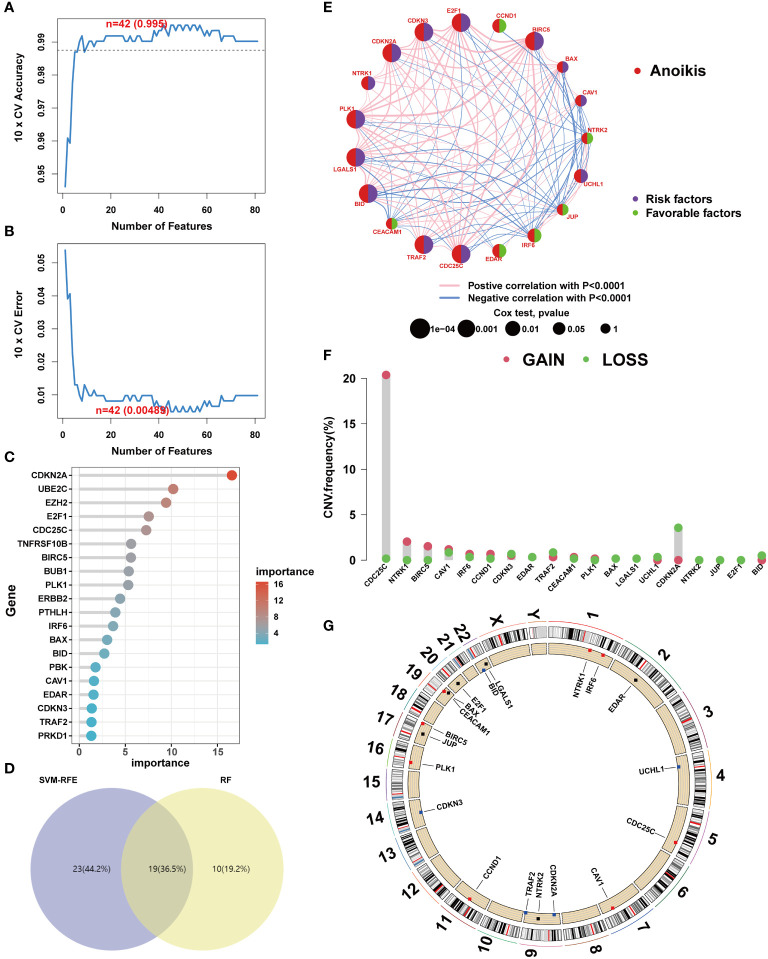
Core anoikis genes screening. **(A, B)** SVM–RFE screens the important anoikis ARGs. **(C)** Top 20 important ARGs. **(D)** Venn diagram. **(E)** ARGs interactions and correlations. **(F)** CNV frequency. **(G)** ARGs’ location in chromosome.

To elucidate the genetic underpinnings of ccRCC, a variety of machine learning techniques were employed for gene screening. Initially, the SVM-RFE algorithm was utilized to validate the screening of candidate genes following a 5-fold cross-validation process (**Figures 3A, B**). Subsequently, an ensemble RF algorithm pinpointed feature genes with a significance threshold exceeding 2, with CDKN2A exhibiting the highest importance (**Figure 3C**). An intersection of candidate genes identified by both SVM-RFE and RF algorithms highlighted 19 ARGs significantly impacting the prognosis of ccRCC patients (**Figure 3D**). In pursuit of understanding the relationships among these pivotal genes, correlation analyses were conducted. The results indicated that BID and PLK1 are markers of poor prognosis in ccRCC patients, and most ARGs demonstrated synergistic interactions (**Figure 3E**). The role of mutations in tumorigenesis was also investigated, particularly focusing on CNV mutation frequencies. Interestingly, a significant gain was only observed in CDC25C, suggesting that mutations might not be the primary mechanism influencing anoikis resistance (**Figures 3F, G**).

### 3.3 ARGs unsupervised cluster analysis

**Figure 4:**
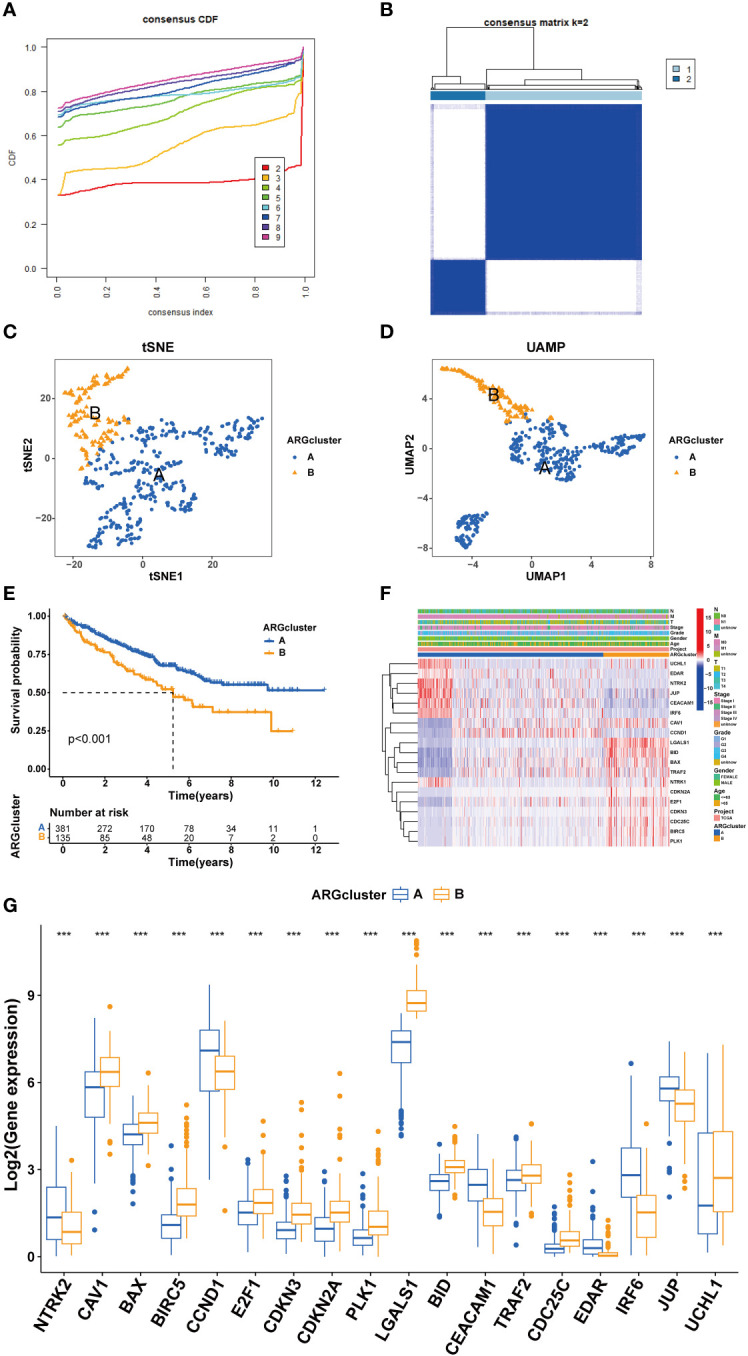
Cluster analysis for ccRCC patients. **(A, B)** cRCC patients were classified into two clusters based on ARGs profiles. **(C, D)** UAMP and tSNE analyses. **(E)** Survival analysis. **(F)** Heatmap based on ARGs and clinical characteristics of ccRCC patients. **(G)** ARGs expression between different clusters. (***P < 0.001).

Clustering of ccRCC patients was performed based on the expression levels of 19 ARGs. When k equals 2, the patients were effectively stratified into two distinct groups (**Figures 4A, B**). Validation of clustering efficacy was provided by UMAP and tSNE scores (**Figures 4C, D**). Survival analysis indicated significant prognostic differences between these two subgroups of ccRCC (**Figure 4E**), with Group A exhibiting superior overall survival (OS) compared to Group B. An examination of clinical data revealed distinct ARG expression patterns and staging characteristics between the subgroups, where higher ARG expression was associated with advanced pathological stages (**Figures 4F, G**).

### 3.4 Development and validation of a risk prognostic model for ccRCC patients

**Figure 5:**
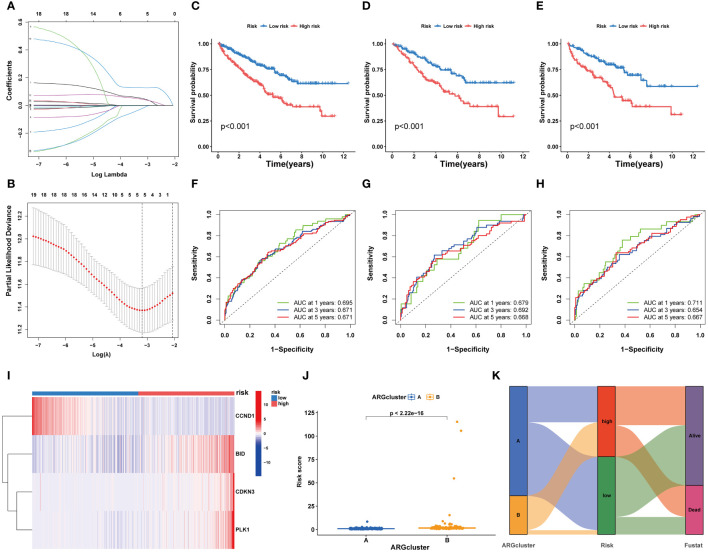
Development and validation of a risk prognostic model for ccRCC patients. **(A, B)** Lasso Cox regression analysis. **(C-E)** K-M curves of ccRCC patients under high and low risk. **(F-H)** ROC curves of ccRCC patients under high and low risk. **(I)** Heatmaps exhibit 4 core ARGs expression patterns. **(J)** The riskscore levels in two ARGclusters. **(K)** Alluvial plots.

Utilizing LASSO Cox regression and multivariate analysis, four core ARGs were identified from the 19 ARGs, and a prognostic model termed ARGs-Riskscore was established (**Figures 5A, B**). This model assigns a specific risk coefficient to each anoikis-related gene to calculate the riskscore, categorizing patients into high-risk and low-risk groups (**Figure 5C**). Kaplan-Meier curves demonstrated poorer survival outcomes for the high-risk group compared to the low-risk group (**Figures 5D, E**). ROC curve analysis revealed that the model’s AUC value exceeded 0.6, indicating substantial accuracy (**Figures 5F–H**). Furthermore, in the high-risk group, the expression levels of CDKN3, PLK1, and BID were elevated, whereas CCND1 showed higher expression in the low-risk group (**Figure 5I**). A higher ARG expression level corresponded to an increased riskscore (**Figures 5J, K**).

Further, we integrated the risk associated with ARGs and clinical data from patients with ccRCC to develop a nomogram that estimates survival probabilities based on age, stage, and risk scores (**Supplementary Figure S1A**). Calibration plots confirmed the nomogram’s accuracy in predicting 1-year, 3-year, and 5-year overall survival (OS) rates (**Supplementary Figure S1B**). Analysis of hazard ratios indicated a strong correlation between age, cancer grade, risk scores, and tumor stage with OS (**Supplementary Figure S1C**). Furthermore, analysis of cumulative hazards revealed that patients with ccRCC who had higher nomorisk scores exhibited increased mortality risks (**Supplementary Figure S1D**).

### 3.5 Anoikis affects tumor immune microenvironment

**Figure 6:**
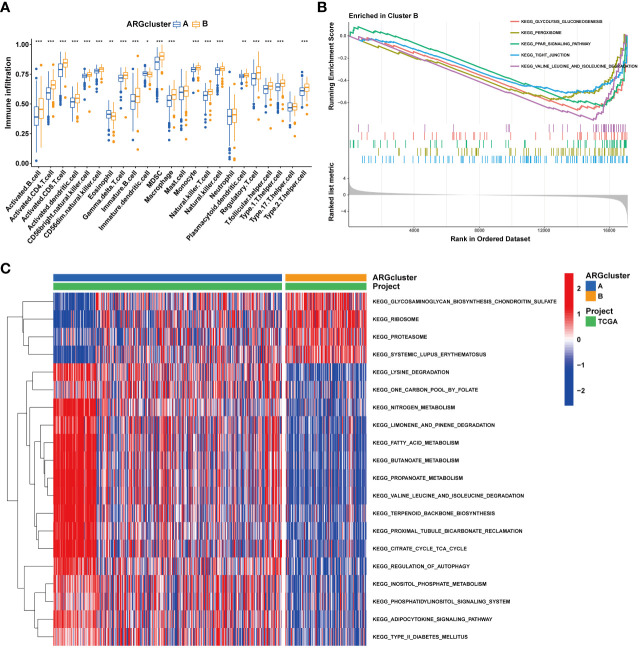
Immune landscape of two ARGcluster. **(A)** Immune cell infiltration levels at two ARGcluster patients. **(B)** GSEA analysis of the enrichment of ARGs. **(C)** GSVA enrichment analysis. (*P < 0.05; **P < 0.01; ***P < 0.001).

**Figure 7:**
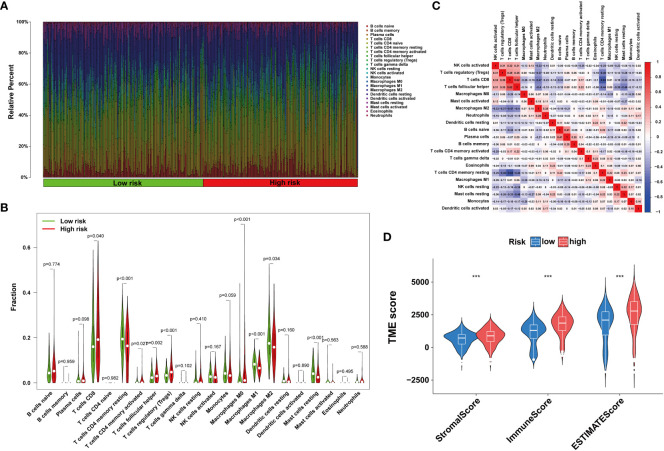
Immune infiltration in under different risk ccRCC patients **(A, B)** Immune cells infiltration analysis. **(C)** Correlation of 23 immune cells. **(D)** TME score. (Wilcox test, ***P < 0.001).

The tumor immune microenvironment plays a pivotal role in the immune evasion processes of cancer. The onset of anoikis resistance is predicated on achieving immune escape. Consequently, we divided ccRCC patients into two subgroups based on the expression patterns of anoikis genes. The findings revealed that patients with high expression of anoikis genes exhibited significantly higher levels of immune infiltration, particularly with MDSC cells (**Figure 6A**), underscoring a close association between the anoikis process and the immunosuppressive microenvironment. Enrichment analyses indicated that, compared to cluster A, the tight junction and PPAR signaling pathways were significantly enriched in cluster B (**Figures 6B, C**), suggesting their major roles in shaping the immunosuppressive microenvironment. Patients with ccRCC of different risk levels demonstrated markedly distinct survival outcomes. Therefore, we further examined the levels of immune cell infiltration in patients classified into high-risk and low-risk groups. It was observed that the proportions of immune cell infiltration varied between different risk groups of ccRCC patients (**Figure 7A**). Notably, Tregs and macrophages M0 were significantly more prevalent in patients at higher risk (**Figure 7B**). Interestingly, there was a significant negative correlation between the infiltration of macrophages M0 and CD8 T cells, and between Tregs and memory CD4 T cells (**Figure 7C**), highlighting the crucial impact of the immunosuppressive microenvironment on anoikis resistance (**Figure 7D**).

**Figure 8:**
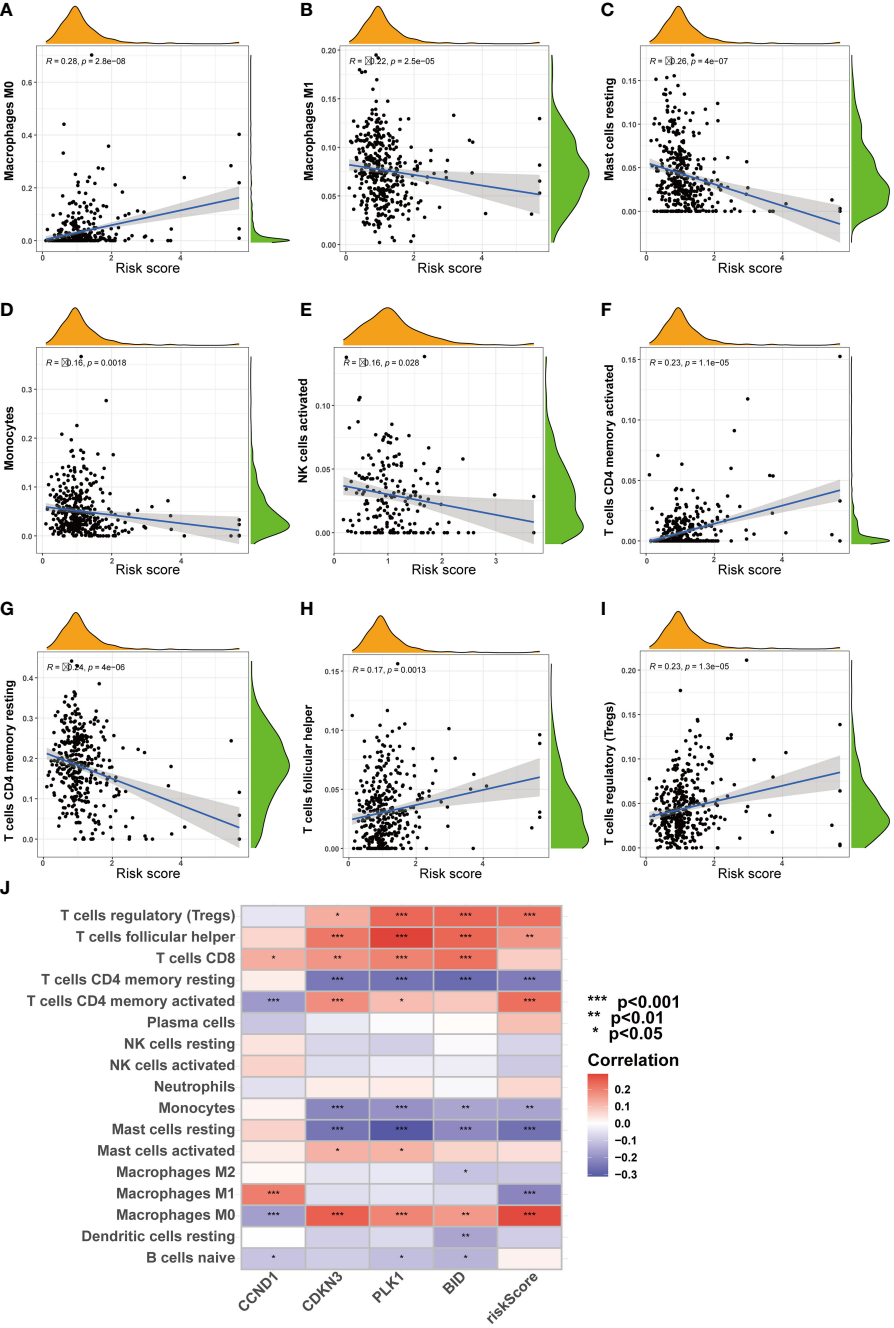
Correlation analysis of riskscore and immune cells. **(A-I)** Correlation of riskscore and immune cells. **(J)** Correlation of four ARGs and immune cells. (*P < 0.05; **P < 0.01; ***P < 0.001).

Furthermore, as risk scores increased, changes were noted in the patterns of immune cell infiltration (**Figure 8J**). For example, the infiltration levels of macrophages M0 and Tregs gradually increased with rising risk scores (**Figures 8A, F, H, I**). In contrast, other cell types, such as macrophages M1 and NK cells, showed a decrease in infiltration as risk scores increased (**Figures 8B–E, G**).

### 3.6 Single-cell analysis reveals anoikis expression pattern in ccRCC

**Figure 9:**
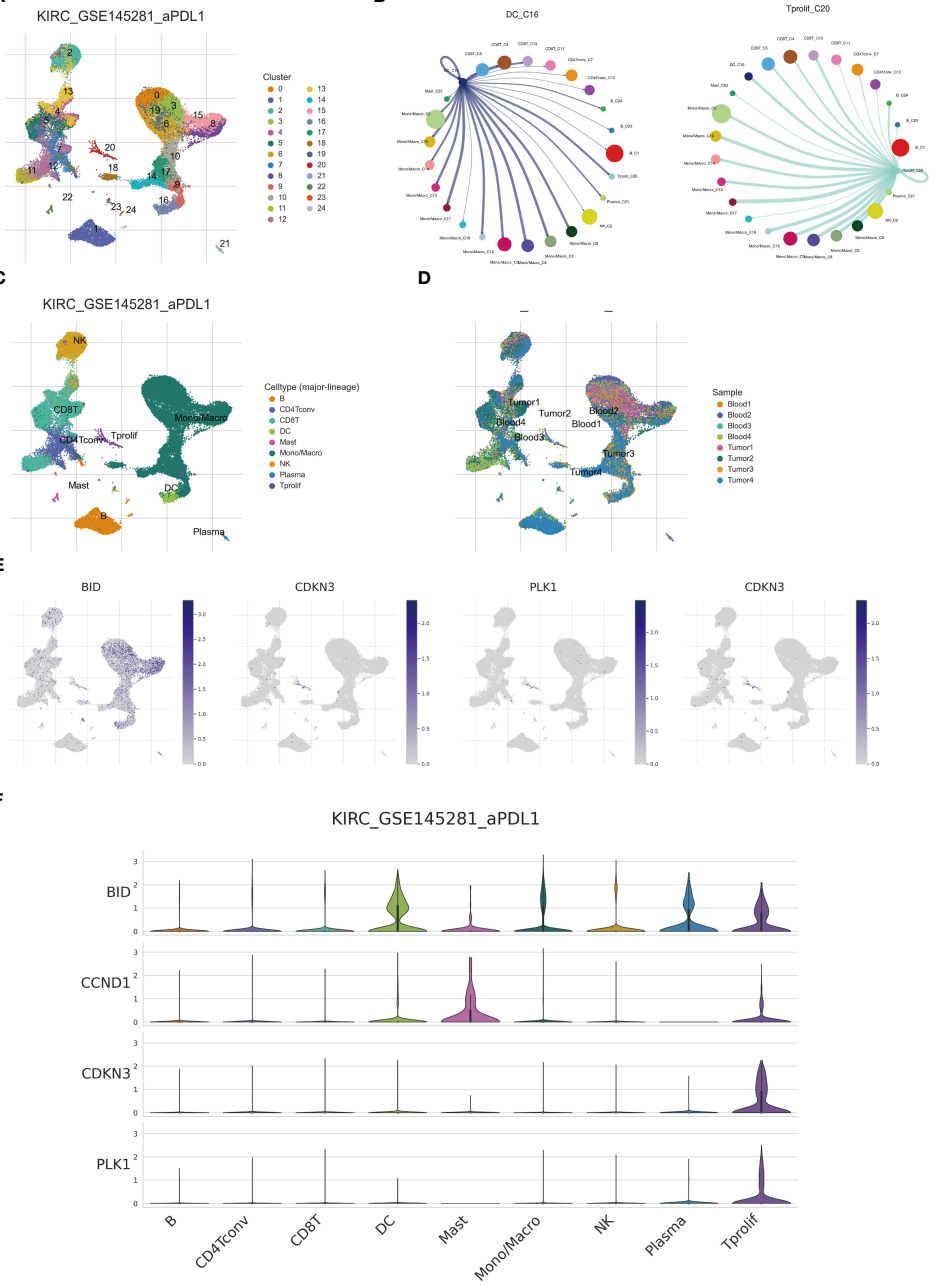
Single cell analysis reveals anoikis genes expression. **(A, C)** Umap of single cell clusters. **(B)** Cell communications of DCs and T proliferation cells. **(D)** Cell types distribution in each ccRCC sample. **(E, F)** 4 core ARGs expression patterns in immune cells.

The advent of single-cell technologies has provided a crucial avenue for exploring cellular subtypes. Utilizing single-cell analysis and annotation, we categorized cell suspensions from patients treated with anti-PD-L1 into 24 immune cell subtypes and nine principal cell types (**Figures 9A, C**). Importantly, the proportions of cellular components from samples of different patients demonstrated notable disparities. For instance, the Tumor 1 sample predominantly consisted of mono/macrophages, whereas CD8 T cells predominated in Blood4 (**Figure 9D**). We further elucidated the expression and distribution of four core ARGs constituting a prognostic model across various cell subtypes (**Figure 9E**). Our findings reveal that BID exhibits the highest expression in DC and T proliferation cells, with subsequent analyses revealing enhanced communication between DC, proliferative T cells, and other cell subtypes (**Figures 9B, F**).

### 3.7 Evaluation of anoikis gene expression by realtime PCR and IHC

**Figure 10:**
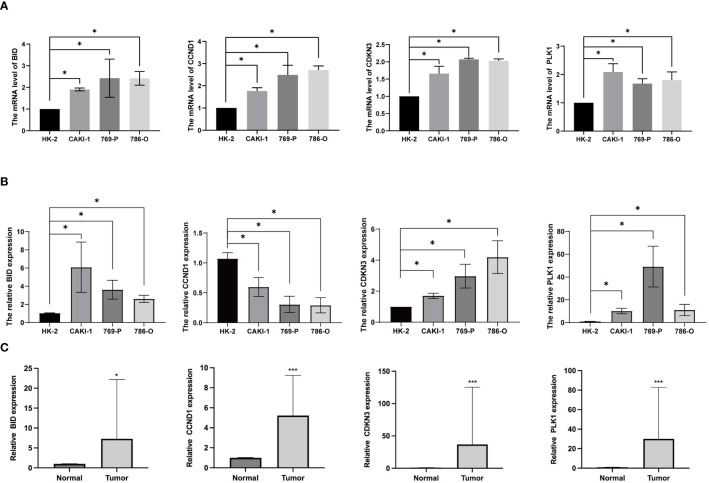
Anoikis genes’ expression levels in ccRCC cells and tissues. **(A)** ARGs mRNA expression levels in primary ccRCC cells. **(B)** ARGs mRNA expression levels in ccRCC cells after culturing for 5 weeks **(C)** The mRNA levels of ARGs in ccRCC and normal kidney tissues. (*P < 0.05; ***P < 0.001).

**Figure 11:**
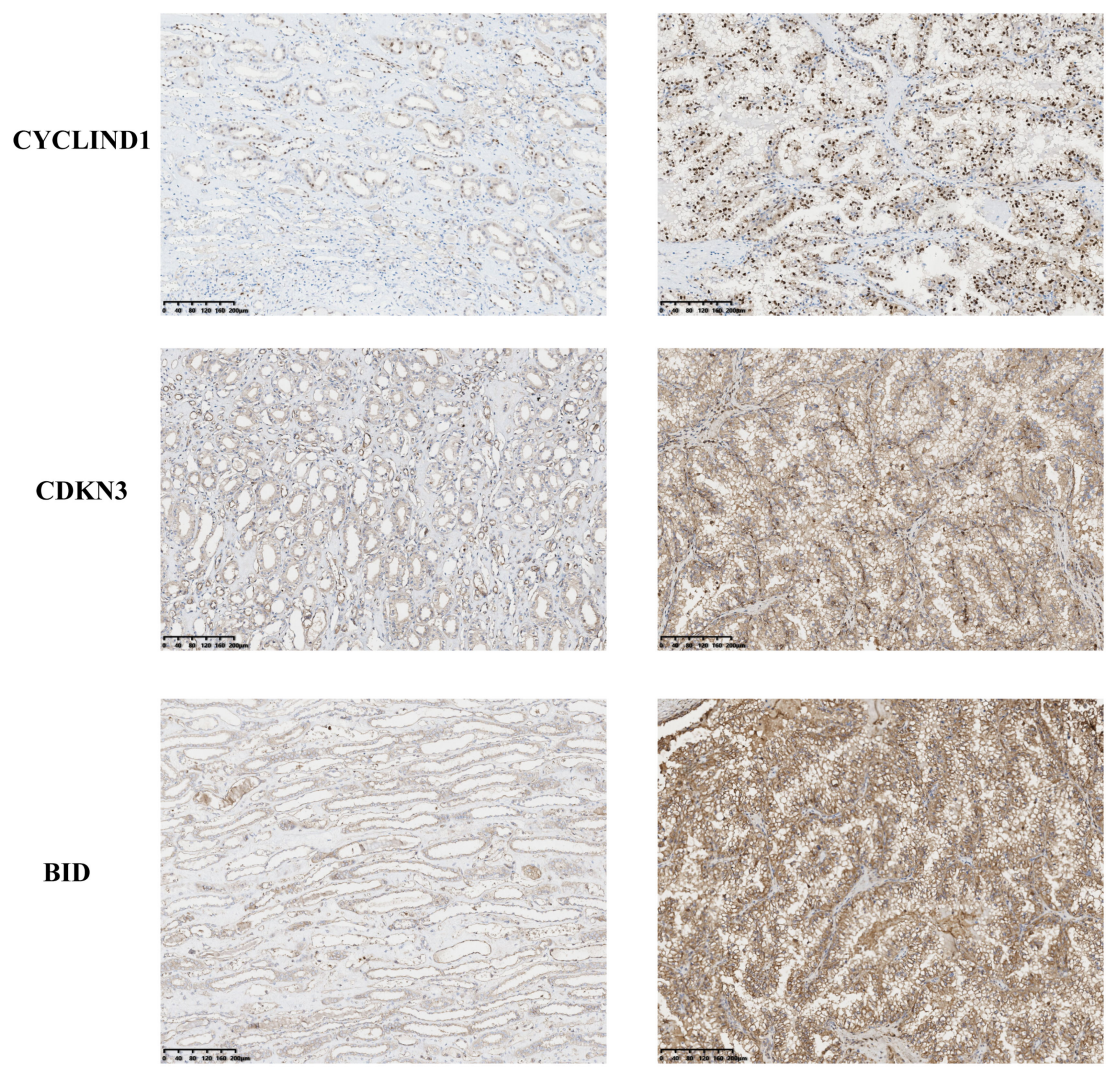
Immunohistochemical results of ARGs proteins.

To investigate the expression of anoikis genes, primary renal carcinoma cell lines and normal renal cell lines were cultured, and the expression levels of four central anoikis resistance genes (ARGs) were compared between these two cell types. The primary cell lines were assessed to ensure the reliability of the results (**Figure 10A**). Furthermore, upon culturing the cells up to the tenth passage, mRNA levels were re-evaluated (**Figure 10B**). Intriguingly, despite the expressions of BID, CDKN3, and PLK1 being consistently higher in the renal carcinoma cell lines than in the normal renal cells across both the primary and tenth passages, shifts in the gene expression levels among the carcinoma lines were noted. Notably, the expression pattern of CCND1 demonstrated an inverse trend. Subsequent analyses involved examining the expression levels of these four core ARGs in cDNA extracted from normal renal tissues and renal carcinoma tissues. The results indicated a higher expression of all four ARGs in the carcinoma tissues (**Figure 10C**). Immunohistochemistry confirmed that the protein levels of ARGs corresponded with the trends observed at the gene expression level (**Figure 11**).

### 3.8 Potency of the anoikis signature in modulating drug resistance

In order to elucidate the association between the anoikis-related signature and drug responsiveness, the IC50 indices for various medications in ccRCC were evaluated (**Supplementary Figure S2**). This analysis implies that individuals with renal carcinoma who are categorized within the high-risk group could exhibit resistance to both chemotherapy and immunotherapeutic approaches. Conversely, this suggests opportunities for modulating drug efficacy through targeted interventions.

## 4 Discussion

As the complexity and diversity of clear cell renal cell carcinoma (ccRCC) become increasingly apparent (39), numerous therapeutic strategies have been introduced into clinical settings to address this condition. Cellular molecular-targeted therapy is the most effective method of treating metastatic ccRCC as patients suffering from kidney cancer do not respond to radiotherapy and chemotherapy. The European Urology Association (EUA) and the United States National Comprehensive Cancer Network (NCCN) recommended the molecular-targeted drugs as the first and second-line medicine for metastatic ccRCC. At present, there are no universally accepted and reliable predictors for the diagnosis and prognosis of ccRCC. The challenge of accurately predicting outcomes persists, highlighting the critical need for the discovery of new biomarkers. These biomarkers are crucial for enhancing the prognosis of ccRCC (40). The exploration of abnormally expressed genes in ccRCC tissues can potentially help identify new molecular biomarkers for the diagnosis and prognosis of ccRCC. Central to this endeavor is anoikis, a cellular process essential for controlling tumor proliferation, spread, and future outcomes (41, 42). Research has linked the development, advancement, and prognosis of ccRCC to specific genes involved in anoikis (43). The goal of this discussion is to integrate findings on the connection between genes related to anoikis and the prognosis of ccRCC, examining their potential relevance in clinical practice and providing a novel theoretical and practical framework for tailored therapeutic approaches.

In this study, we identified critical roles of anoikis-associated genes in ccRCC and developed a predictive model. Herein, we described the differential expression of anoikis-associated genes in tumor tissues relative to normal samples and investigate the potential regulatory role of anoikis-associated genes in controlling the ccRCC immune microenvironment. In addition, we investigated the relationship between anoikis-associated genes expression levels and immunotherapy. An analysis of 161 anoikis-associated genes revealed four that were conclusively linked to the prognosis of ccRCC. Our study confirmed that BID, CCND1, CDKN3, and PLK1 showed upregulation in ccrCC tissues, with significantly higher expression compared to normal cells. It is reasonable to speculated that these four genes play critical roles in ccRCC tumorigenesis and progression. This study aimed to gain insights into the underlying mechanisms associated with the anoikis-associated gene that was associated with immune-related factors. BID, a pro-apoptotic protein in the Bcl-2 family, functions collaboratively with BAX to facilitate cellular apoptosis (44). Research by Ji Miao and colleagues showed that the expression levels of Bid correlate with the susceptibility of liver cancer cells to chemotherapeutic agents (45). Another key protein, Cyclin D1 (CCND1), a crucial component of the D-type cyclin group, regulates the progression of the cell cycle (46). Recent studies suggested the USP10/CCND1 pathway as a potential therapeutic target for glioblastoma (GBM) patients (47). Investigations by Hongying Zhang and team found that CCND1 suppression, achieved by gene silencing, impedes the differentiation of hepatic cancer stem cells by inhibiting autophagy (48). Furthermore, the expression patterns of CCND1 are strongly correlated with the initiation and progression of multiple cancer types (49–51).

CDKN3, a cyclin-dependent kinase inhibitor, is identified as a crucial therapeutic target for cervical cancer (52) and plays a role in the malignant advancement of pancreatic cancer by interacting with PSMD12 (53). Its expression in various cancers modulates resistance to treatment. Aolin Li and colleagues demonstrated that ZNF677 represses the malignant evolution of renal cell carcinoma through the reduction of CDKN3 expression (54). Furthermore, the circular RNA circSDHC binds to miR-127–3p competitively, thereby diminishing CDKN3 expression in renal cell carcinoma and curbing its malignant advancement (55). These observations corroborate our analysis, thereby confirming the precision of our findings.

Polo-like kinase 1 (PLK1), an eminent serine/threonine kinase within the protein kinase superfamily, promotes the advancement of mitosis (56). Elevated levels of PLK1 are commonly observed in cancerous tissues, highlighting its potential as a target for therapeutic intervention (57). Suppression of PLK1 enhances the response of pancreatic cancer cells to immunotherapeutic strategies (58). Likewise, a reduction in PLK1 activity increases the sensitivity of breast cancer to radiation therapy (59), whereas enhanced expression of PLK1 contributes to the development and advancement of liver tumors (60).

Infiltration of Treg cells is frequently associated with poorer prognoses across various cancers, and a reduction in Treg cells has been observed to initiate and enhance antitumor immune responses. In this study, the risk score exhibited a significant positive correlation with the level of Treg cell infiltration, whereas an inverse trend was noted for NK cells, suggesting a critical role for the immunosuppressive microenvironment in anoikis resistance, subsequently impacting the overall survival of patients with ccRCC. The signaling cascade mediated by PPAR, documented to enhance angiogenesis within tumor matrices (61), is associated with the pathogenesis of both inflammatory and neoplastic conditions (62). Furthermore, this pathway has been shown to trigger anoikis in certain cell types under *in vitro* conditions (63). Our findings suggest that the PPAR pathway may serve as a primary mechanism by which Tregs orchestrate an immunosuppressive microenvironment, thereby facilitating anoikis resistance, which in turn supports distant metastasis and immune evasion in ccRCC. Conventional surgical treatment and radiotherapy and chemotherapy cannot be effective to treat patients suffering from late-stage ccRCC. Maybe more research should be conducted on the gene targets and immune checkpoint inhibitors associated with ccRCC as the results can potentially help predict the prognosis of antitumor immunotherapy. It is worth noting that the results of our research reflected the association of anoikis-associated genes with a substantial prognosis of ccRCC and confirmed the reliability of the analytical results obtained. We may infer that the modulation of the Anoikis-associated genes activity associated with ccRCC could potentially help obtain results that can help improve the therapeutic techniques.

As we know, there is a minor number of relevant researches currently available to explain the functions of anoikis in ccRCC. Our work provided valuable information on how the anoikis-associated gene participated in cancer immunotherapy, which may potentially help improve the processes of ccRCC targeting therapy. In our next step, we need to extend the existing database and mutually authenticate to larger database. Experiments should be performed at the molecular, cytological, and animal levels to investigate the relationship between the prognosis of the patients and the properties of the clinical tumor tissue samples. We believe that our results can potentially help for improving the efficiency of diagnosis, treatment methods, and survival prognosis of ccRCC patients.

## 5 Conclusion

In this study, we established the association between anoikis, immune cell infiltration, and the prognosis of clear cell renal cell carcinoma (ccRCC) patients through multi-omics and single-cell analyses. Furthermore, we elucidated their impact on the efficacy of immune therapy. These findings not only provide novel insights into the role of apoptosis in cancer progression but also highlight new research directions for immunotherapeutic strategies in ccRCC.

## Data availability statement

The original contributions presented in the study are included in the article/**Supplementary Material**. Further inquiries can be directed to the corresponding author.

## Ethics statement

The studies involving humans were approved by Ethics Committee of University of Hong Kong Shenzhen Hospital. The studies were conducted in accordance with the local legislation and institutional requirements. The participants provided their written informed consent to participate in this study.

## Author contributions

XW: Data curation, Formal analysis, Investigation, Methodology, Software, Validation, Visualization, Writing – original draft. JH: Formal analysis, Methodology, Software, Visualization, Writing – original draft. TQ: Methodology, Software, Visualization, Writing – original draft. XC: Data curation, Resources, Visualization, Writing – original draft. GL: Formal analysis, Investigation, Writing – original draft. SF: Formal analysis, Investigation, Writing – original draft. SX: Formal analysis, Resources, Writing – original draft. LQ: Formal analysis, Resources, Writing – original draft. WW: Conceptualization, Funding acquisition, Investigation, Project administration, Resources, Supervision, Validation, Writing – review & editing.

## Supplementary material

Supplementary Figure 1 Nomogram establishment based on risk and clinical information. **(A)** A monogram graph to predict ccRCC patients’ prognosis. **(B)** Calibration plots. **(C)** Multivariate analysis. **(D)** Cumulative risk values analysis. (**P* < 0.05; ****P* < 0.001).

Supplementary Figure 2 Drug sensitivity analysis.
