# Supplementary figures and images for: Anoikis resistance regulates immune infiltration and drug sensitivity in clear-cell renal cell carcinoma: insights from multi omics, single cell analysis and in vitro experiment
Source: Front Immunol. 2024 Jun 17;15:1427475. doi: 10.3389/fimmu.2024.1427475 (PMC11215044; doi:10.3389/fimmu.2024.1427475)

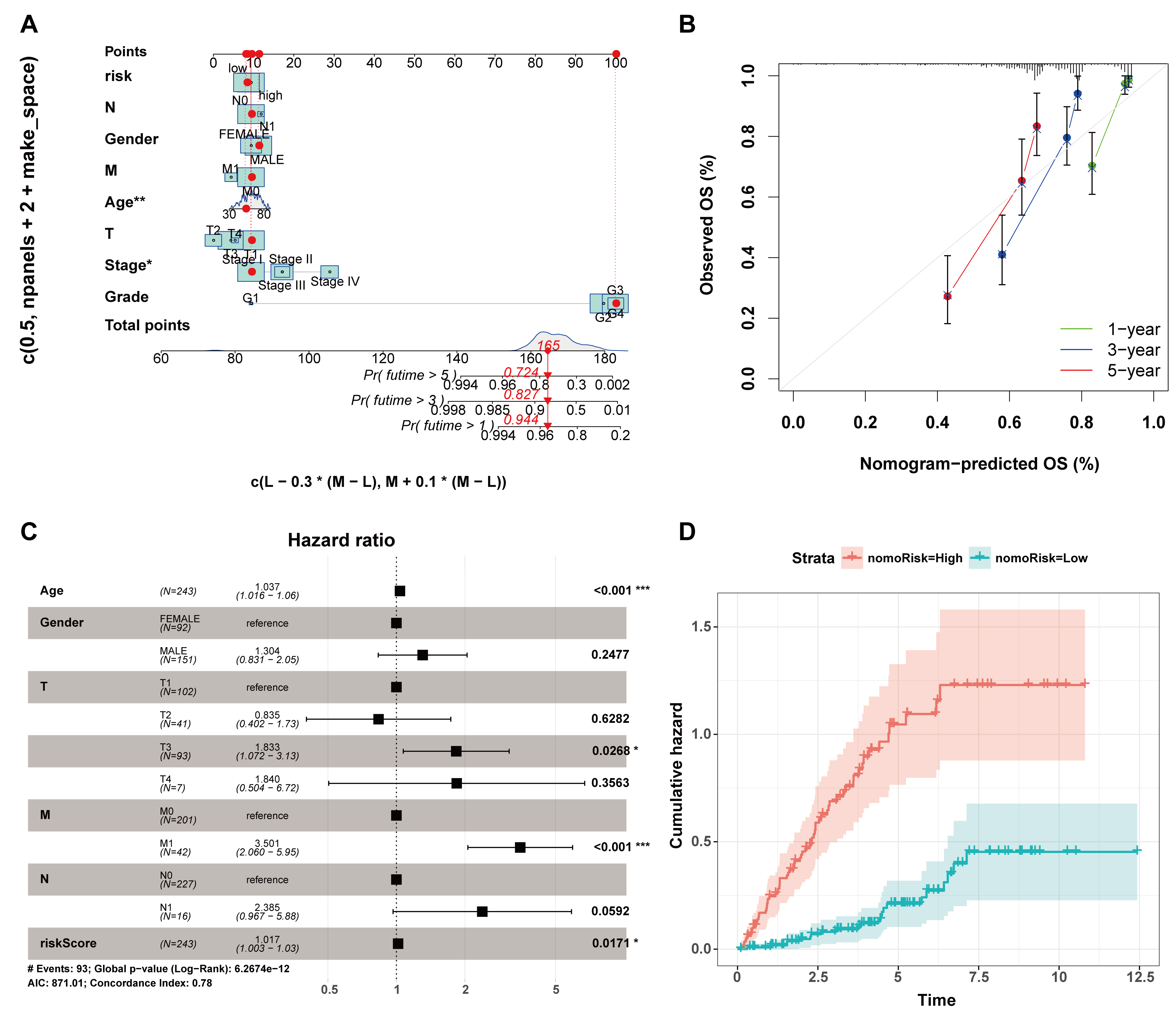

Supplement: Supplementary Figure 1 — Nomogram establishment based on risk and clinical information. (A) A monogram graph to predict ccRCC patients’ prognosis. (B) Calibration plots. (C) Multivariate analysis. (D) Cumulative risk values analysis. (*P < 0.05; ***P < 0.001). [file Image_1.tif]

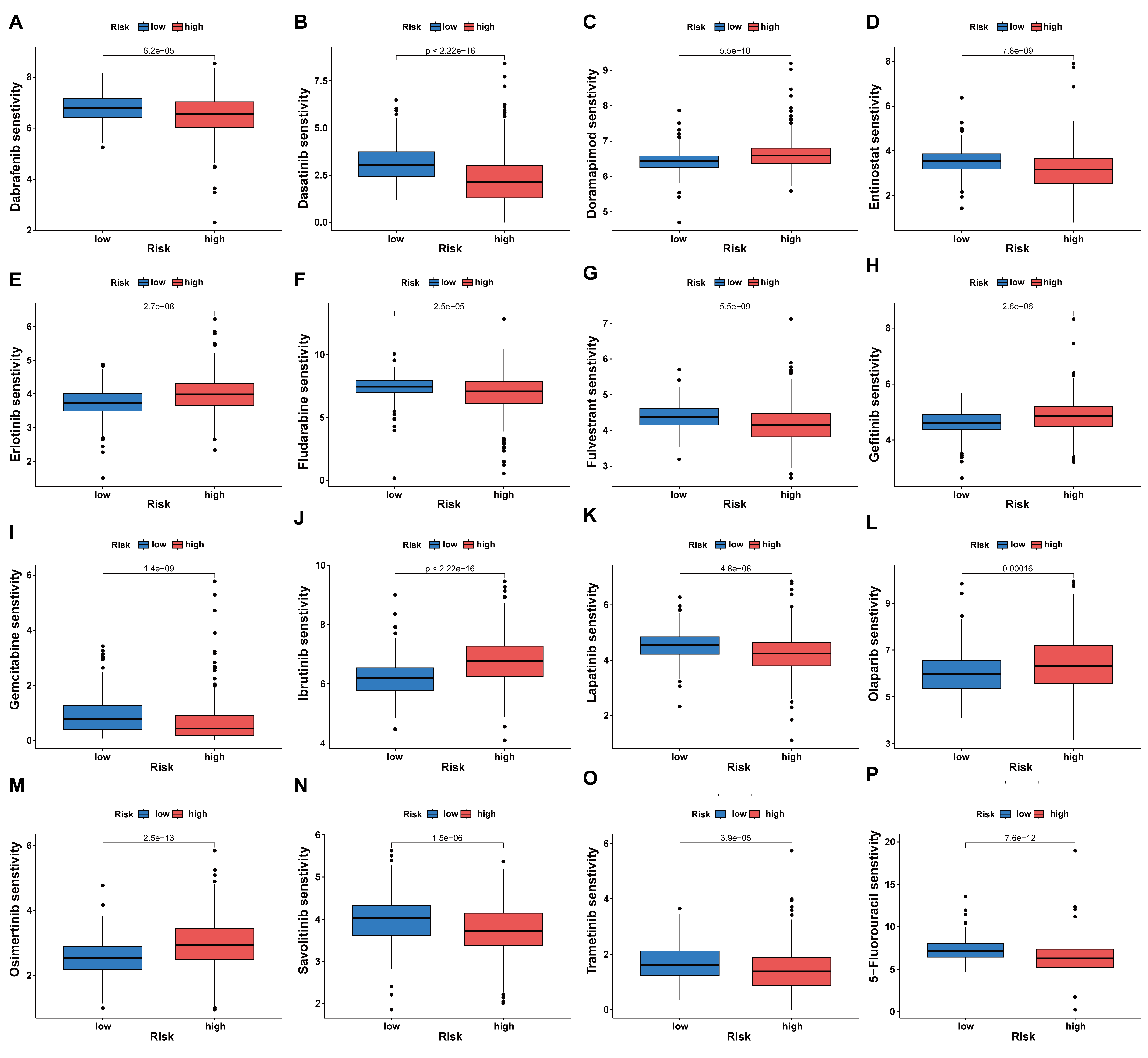

Supplement: Supplementary Figure 2 — Drug sensitivity analysis. [file Image_2.tif]
